# The New Mental Deficiency Bill

**Published:** 1913-05-31

**Authors:** 


### The Hospital

The Workers' Newspaper of Administrative Medicine and Institutional Life, Administration, National Insurance and Health.

No. 1405, Vol. LIV SATURDAY, MAY 31, 1913. PRICE ONE PENNY.

### THE NEW MENTAL DEFICIENCY BILL.

A consideration of the new Mental Deficiency Bill shows that, as compared with the Bill of 1912, the Government have made considerable changes on some very important points. The clauses defining the persons who are defectives within the meaning of the Bill have been considerably modified. The definitions which are now given of the various classes of defectives are practically those which were agreed upon by the Standing Committee of the House of Commons. The present Bill will not arouse such violent antagonism nor will it elicit such enthusiastic support. With one exception it is for practical purposes a better Bill. Those who are keenly concerned with the safeguarding of feeble-minded and morally imbecile girls will not be satisfied with its provisions. There are two extremes from which this aspect of the grave problem is considered. On the one hand are those to whom the liberty of the subject is as the Ark of the Covenant, and they are always in dread lest a profane hand is about to commit sacrilege. In these days of compulsory insurance, and when a man may not work for the support of his wife and family unless in keeping with the orders of a trade union, it is delightful to meet with men who still retain, in their full freshness, such enthusiasms. They cannot hear of homes for the feeble-minded being provided without at once recalling all the horrors of "lunatic asylums" depicted by Charles Reade. Men who hold these extreme views are sincere, and we could not afford to be without them. If comparatively few in number they are insistent on being heard. On the other side is the social worker, who has been daily brought face to face with a state of things which produces and tends to multiply misery, disease, and moral degradation. To him the risk of any danger to the liberty of the subject is a small thing in comparison with the magnitude of the evil he sees spreading before him. His familiarity with the working of the Poor Law and the Lunacy Acts teaches him to think lightly of the warnings as to the liberty of the subject. He feels that the evil is so great that it must be dealt with.

The time has come when our attitude towards the extreme view of the enthusiastic advocate of personal liberty must be one of respectful disregard. We have before us as we write the details of a family history. The grandmother was insane and died in a mental hospital. Two daughters were feeble-minded and certifiable. They have six illegitimate children between them and they are all mentally defective; two are certified. The existence of such offspring is in itself a damning indictment of our present want of system in dealing with this aspect of the question. Every mental hospital could give similar examples. We confess that our judgment and our sympathies lead us to support the social worker, who says that the time has come when effective steps must be taken to stop this evil. The proposals of the Government cannot be called too drastic. We fear they do not go far enough. The most dangerous cases are not caught by the Bill. For their own sakes and in the interest of the nation at large this is deeply to be regretted.

It was wise not to complicate the Bill by the inclusion of cases of mental infirmity due to old age. They stand on a different footing. Their presence would have changed the character of institutions to which feeble-minded might be sent and made them difficult to distinguish from mental hospitals for chronic cases. It would be well if there were a simpler means of interchange between the mental hospitals and the homes for feeble-minded. They might, while separate, be able to work together.

Hope for the future lies in the cordial interworking of Education Committees with the local Committees appointed to administer the Act. Those enlightened bodies which have already taken advantage of the permission to establish special schools for defectives may congratulate themselves on the Elementary Education (Defective and Epileptic Children) Bill, which has been introduced by the President of the Board of Education. This makes it compulsory for local authorities to make provision for the education of mentally defective children from the age of seven. It would be well if it made the Act of 1899 compulsory also in relation to epileptic cases. It is estimated that there are over fifty thousand mentally defective children in England and Wales who ought to be in special schools. The extent to which the permission to establish such schools has been taken advantage of will be understood when we say that over forty thousand are still without any such provision for their education. We do not think the public have any conception of the amount of money that will be required to provide for the proper care and control of our defectives. The sum provided under the Bill of the Government may do for a beginning. It will be found to be absurdly inadequate. It will, however, in the future prove to be a good investment from the point of view of the ratepayer.

